# Maternal depression and child development at 3 years of age: a longitudinal study in a Brazilian child development promotion program

**DOI:** 10.1038/s41390-023-02876-9

**Published:** 2023-11-11

**Authors:** Iná S. Santos, Cauane Blumenberg, Tiago N. Munhoz, Alicia Matijasevich, Cristiane Salum, Hernane G. Santos Júnior, Letícia Marques dos Santos, Luciano L. Correia, Marta Rovery de Souza, Pedro I. C. Lira, Caroline C. Bortolotto, Raquel Barcelos, Elisa Altafim, Marina Fragata Chicaro, Esmeralda Correa Macana, Ronaldo Souza da Silva

**Affiliations:** 1 Programa de Pós-Graduação em Epidemiologia, Universidade Federal de Pelotas, Pelotas, RS Brazil; 2 Causale Consultoria, Pelotas, Brazil; 3 Curso de Psicologia, Universidade Federal de Pelotas, Pelotas, RS Brazil; 4 Departamento de Medicina Preventiva, Faculdade de Medicina FMUSP, Universidade de São Paulo, São Paulo, SP Brazil; 5 Centro de Matemática, Computação e Cognição, Universidade Federal do ABC, Santo André, SP Brazil; 6 Instituto de Saúde Coletiva, Universidade Federal do Oeste do Pará, Santarém, PA Brazil; 7 Instituto de Humanidades Artes de Ciências da Universidade Federal da Bahia (UFBA), Salvador, BA Brazil; 8 Departamento de Saúde Comunitária, Universidade Federal do Ceará, Fortaleza, CE Brazil; 9 Departamento de Saúde Coletiva, Universidade Federal de Goiás, Goiânia, GO Brazil; 10 Departamento de Nutrição do Centro de Ciências da Saúde, Universidade Federal de Pernambuco, Recife, PB Brazil; 11 Programa de Pós-Graduação em Saúde Mental, Faculdade de Medicina de Ribeirão Preto, Universidade de São Paulo, Ribeirão Preto, SP Brazil; 12 Fundação Maria Cecília Souto Vidigal, São Paulo, SP Brazil; 13 Itaú Social, São Paulo, SP Brazil; 14 Secretaria de Avaliação e Gestão da Informação (SAGI), Ministério da Cidadania, Brasília, DF Brazil

## Abstract

**Background:**

We tested the hypothesis that children of non-depressed mothers perform better in a developmental test at 3 years than children of depressed mothers.

**Method:**

Longitudinal analysis from a trial to assess the impact of a child development promotion program in 30 Brazilian municipalities. Mothers and children were appraised at first-year post-partum, 1 and 3 years after enrollment. Child development was assessed through the Ages and Stages Questionnaire (ASQ3) and maternal depression through the Edinburgh Postnatal Depression Scale (EPDS). Crude and adjusted beta coefficients were obtained by linear regression before and after multiple imputation.

**Results:**

In total, 2098 mother/child dyads were included and 8.2% of the mothers had persistent depressive symptoms. There was a decrease in ASQ3 as the number of follow-ups with EPDS ≥ 10 increased (*p* for trend <0.001). In adjusted analysis, the direction of the association persisted but lost statistical significance. After multiple imputation, children from mothers with EPDS ≥ 10 in three follow-ups presented a decrease of about 14 points in ASQ3 (adjusted beta coefficient = −13.79; −22.59 to −5.00) (*p* for trend = 0.001).

**Conclusions:**

Identification of women at increased risk of depression should be among the primary health care sector priorities in maternal and child health in Brazil.

**Impact:**

In our population study, almost one in every ten women presented persistent depression symptoms across the first 3 years postpartum. In adjusted analysis there was a detrimental impact of persistent maternal depression on child development at 3 years of age. The persistent exposure to maternal depression across early childhood negatively influences children’s development. Considering its prevalence, identification of women at increased risk of depression should be among the primary health care sector priorities in maternal and child health in Brazil.

## Introduction

The time from pregnancy to age 3 years is the most critical for brain development, with ~80% of a baby’s brain formation occurring during this window.^1,2^ Life experiences up to age 5 years provide the foundation for future life success, and failure to foster cognitive skills (age-related increases in language, intellectual, and executive functioning capabilities) during this period impairs the child ability to learn at later ages,^3^ thus resulting in social, emotional, and educational functioning deficits into adulthood. Nonetheless, in low- and middle-income countries, close to 250 million children below 5 years are at risk of sub-optimal development due to extreme poverty.^4^

Home visiting programs can have moderate to high positive effects on early childhood development when high-quality implementation processes are achieved.^5,6^ In view of this, in October 2016, Brazil became the first country in the world to launch a national home visiting program aimed at promoting the development of socially vulnerable children—the Happy Child Program (*Programa Criança Feliz*—PCF).^7^ An independent evaluation of the impact of PCF on child development was conducted by means of a randomized controlled trial.^8^ At the endline survey (36 months after enrollment in the study), intent-to-treat analyses and analyses using instrumental variables and propensity scores matching showed no effect of PCF on child development. Previous analyses with data from the evaluation study showed that the prevalence of maternal depression at the first year postpartum as measured by the Edinburgh Postnatal Depression Scale (EPDS)^9^ (EPDS ≥ 10)^10^ was 26.5% (25.0–28.1%),^11^ and that 14.1% (11.3–17.6%) of the women presented persistent depressive symptoms in the second year postpartum.^12^

Studies suggest that maternal depressive symptoms negatively affect children’s cognitive, behavioral, and socio-emotional development.^13^ Nonetheless, the evidence for the association between maternal depression and cognitive development is mixed, because some studies have found associations, whereas others either find no association or only find associations for specific groups of depressed mothers such as those with low levels of education or low socioeconomic status.^14^ The time elapsed between exposure measurement and child development seems to play a role in these inconsistencies. Studies assessing maternal depression over a shorter period of time (first 2 years of life) are less likely to find an association given increasing evidence that it is the persistent exposure to maternal depression across early childhood that negatively influences children’s cognitive development.^15^

Therefore, given that (1) the PCF had no effect over child development, (2) the prevalence of maternal depression in the sample was high, and (3) the potential association between maternal depression and impaired child development,^15^ the current study aimed to test the hypothesis that, in a sample of poor families living in a middle-income country, children of non-depressed mothers would perform better in a developmental test at 3 years of age than children of depressed mothers.

## Methods

This study is a longitudinal observational analysis of the data from the randomized controlled trial carried out to assess the impact of the PCF on child development.^8^

### Happy Child Program (Programa Criança Feliz—PCF)

Children from families enrolled in the *Bolsa Família Program*,^16^ a government cash transfer program that covered 40 million Brazilians, were eligible to PCF. The *Bolsa Família* is delivered to families whose per capita monthly income is less than Brazilian Real (BRL) 85.00 (U$ 16.00); if the family includes children or adolescents up to 17 years of age, this cutoff increases to BRL 170.00 (U$ 32.00) per month. Maintenance of the *Bolsa Família* transfer is conditioned upon some requirements that the family must meet: enrolling and keeping the children and adolescents from 6 to 17 years of age in school and taking children under 7 years of age to health units for immunization and monitoring growth and development, according to the schedule recommended by the health teams. For pregnant women, a further condition is appearing for prenatal appointments.

Specific objectives of the PCF include guiding and supporting pregnant women and their families in preparation for the birth of the child; collaborating in the exercise of parenting; strengthening the roles of families in the care, protection and education of children; and favoring the strengthening of affectional and community bonds. Additional objectives include promoting actions aimed at integral development in early childhood, encouraging the development of recreational activities with the involvement of other family members; promoting and monitoring child development; and facilitating access to other public services. The cornerstone of PCF are weekly visits to the children and their families, starting during pregnancy and continuing until the child reaches 3 years of age. The program does not include any interventions aimed at addressing maternal depression.

### The impact evaluation of PCF on child development

The baseline survey (T0) for the trial was conducted from August 2018 to July 2019, when a total of 3242 children under 12 months of age residing in 30 municipalities from six states of Brazil were enrolled. In each state, three to six municipalities with sufficient numbers of eligible children aged under 12 months were selected. Randomization into intervention and control groups was carried out soon after the baseline interview. The original design of the evaluation study included four visits to all children: the baseline study (referred to as T0), the first-year follow-up study (T1) in late 2019, the second follow-up (T2) in late 2020, and the third and last follow-up (T3) in late 2021. The first follow-up (T1) survey was carried out from September 2019 to January 2020, when the same children were visited at 12–23 months of age. The Covid-19 pandemic led to the cancellation of the T2 visits, and the T3 survey was conducted from October 2021 to January 2022. Subgroup analyses examining the impact of PCF according to maternal depression did not show significant impact of the program. At T3, in both control and intervention groups, children from depressive mothers presented lower ASQ3 scores, but there was no difference between the two groups and the *p* value for interaction between groups (PCF and control) and maternal depression on ASQ3 score was 0.455. Further details on the assessment methodology are available elsewhere.^17^

### Child development

Child development was assessed in the T0, T1 and T3 using the Ages and Stages Questionnaire (ASQ3),^18^ which was validated for use in Brazil.^19–21^ ASQ3 includes 30 items from five domains: cognition (problem solving), communication, fine and gross motor, and personal social. Each domain includes six questions answered by the mother regarding developmental milestones, with three possible replies: not yet (0 point), sometimes (5 points) and yes (10 points). For each age stratum, the maximum score including the 30 questions equals to 300 points.

The items investigated are age specific. The T3 questionnaire included the modules for average ages 36, 42 and 48 months. The 36-month questionnaire evaluates children from 34 months and 16 days to 38 months and 30 days; the 42-month questionnaire assess the children aged 39 months and 0 days to 44 months and 30 days; and the 48-month questionnaire, the children aged from 45 months and 0 days to 50 months and 30 days. The developmental milestones assessed include understanding simple verbal structures; correct use of plurals; coordination of legs and feet for balance and moving; ability to use tools such as knobs, scissors, taps, pencils, and pens; manipulate two pieces of information at the same time; imitate or copy adults; assign meanings; recognize and categorize objects and people; in addition to checking whether the child is independent in daily tasks, such as eating, dressing and cleaning, among other skills.

The standard ASQ3 solely relies on respondent’s report regarding abilities of the child. To increase the objectivity of the results, the research team derived an additional ASQ3 version using exactly the same milestones as the standard version but based on observation of task completion by the interviewers. Each interviewer carried objects such as toys, child clothes, personal hygiene items, paper and pencils, and children were requested to complete each ASQ3 task while being observed. When the child was unable to complete a task, the respondent was asked, and her/his information was recorded. In the current study, the observed score was employed.

### Maternal depressive symptoms

The EPDS explores the presence of common symptoms of depression in the last 7 days before the interview. Each of the ten questions of the scale corresponds to a clinical depressive symptom, such as guilt feeling, sleep disturbance, low energy, anhedonia, and suicidal ideation, with four possible answers, ranging from 0 (never or not at all) to 3 (yes, “as usual”, “very often” or “most of the time”), and with some items having reversed order of scores. Higher scores indicate more depressive symptoms. The scale total score varies from zero to 30. The scale was applied verbally by the interviewer during a single home visit following the order of questions in the instrument. The EPDS has been translated into Portuguese and used in several studies in Brazil.^10^ In the current study, maternal depression was analyzed as the number of follow-ups with EPDS score ≥10 and EPDS score ≥13 (both varying from 0 to 30). Although the EPDS is a screening—rather than diagnostic test, we used the term “depression” interchangeably with an EPDS ≥ 10 and EPDS ≥ 13.

### Potential confounding factors

The family and individual characteristics of the children and their parents collected at T0 were included as potential confounders. Schooling of the child’s mother (0–4, 5–8 and ≥9 years), whether the mother lived with husband or partner (yes/no), and maternal self-reported skin color (White, Brown, Black, or other). Mother’s age (<20, 20–24, 25–29, 30–34, and ≥35 years); planning of the pregnancy (yes/no); number of antenatal care consultations in the pregnancy of the index child (0–5 or ≥6); first antenatal care visit (first, second or third trimester of pregnancy); self-perceived support from the child’s father (yes/no) and the family during the pregnancy (yes/no); and mother working outside the household (yes/no).

The child’s characteristics included sex (female/male); age at the time of T0 interview in months (0–3, 4–6, 7–11); and intra uterine growth generated from the combination of gestational age and birthweight: “term birth with adequate weight for gestational age (AGA)”, “preterm with AGA”, “term, small for gestational age (SGA)”, and “preterm SGA”. SGA was classified as children with weight for gestational age and sex below the 10th percentile of the standard curve.^22^ The number of children under 7 years of age living in the household (including the PCF beneficiary child) was also recorded.

### Statistical analyses

This was a longitudinal analysis of the randomized trial. Initially only women with full information on EPDS scores at T0, T1 and T3, and on ASQ3 score of their children at T3 were entered in the study. The analysis consisted of the sample’s description and the comparison between women and children with complete data and those with missing data (the sample with missing data on EPDS in at least one follow-up and/or missing information on child ASQ3 at T3), followed by calculation of the proportion of women with none, one, two and three follow-ups with EPDS ≥ 10 and EPDS ≥ 13. Tests for trend were carried out after the heterogeneity tests whenever the later were statistically significant (*p* < 0.05). If the test for trend was also statistically significant, we presented the *p* value for the trend test, otherwise we kept the *p* value of the heterogeneity test.

To understand the impact of the missing information on exposure and outcome, we performed sensitivity analyses in which we imputed missing information using a multiple imputation (*M* = 110) with chained equations approach. The following predictors (all measured at T0) were used to impute missing EPDS scores at T0, T1 and T3, and the ASQ3 scores at T3: random allocation of the child, child age, sex and baseline ASQ3 score, maternal schooling, family and child’s father support during pregnancy, number and timing of beginning of antenatal care consultations, parity, maternal age and working outside the household, and number of children under seven living in the household. Follow-up rates of the trial were adequate (80% after 3 years) and similar across subgroups of children and families, indicating low risk of bias due to loss to follow-up,^8^ then missing values were assumed to have occurred at random.

Linear regression was used to obtain crude and adjusted beta coefficients with the respective 95% confidence interval (95%CI). All variables were entered at the adjusted model, and one-by-one those associated with child development at *p* value > 0.20 were removed in backward fashion from the model. Variables associated with child development with *p* ≤ 0.20 were retained in the multivariate model as confounders.^23^ All analyses were multilevel, considering the natural clustering of data by state and municipality of the mother’s residence: the third level consisted of the states of Brazil, the second of the study’s municipalities, and the first of the maternal depression and postulated confounders. Statistical significance was set as two-tailed *p* < 0.05. All the analyses were adjusted for the random allocation of children to the intervention or control groups. Statistical analyses were performed using Stata version 17.0 (StataCorp^®^, College Station, TX).

## Results

**Fig. 1:**
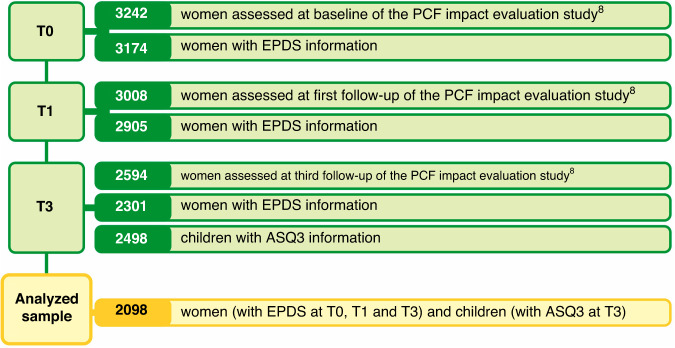
Flow diagram of the study. T0: baseline survey when children were aged <12 months; T1: first year follow-up when children were aged 12–23 months; T3: third follow-up when children were aged 36–47 months.

The flow diagram in Fig. 1 shows that a total of 2098 women had full information on EPDS scores at T0, T1 and T3, and on ASQ3 score of their children at T3, and were included in the analyses.

### Sample description

Table 1 shows that almost half (49.8%) of the analytical sample was composed of women between 20–29 years old, while 13.2% were adolescents, and 15.7% were 35 years or older. Almost three quarters of the women self-reported Brown skin color, the majority had ≥9 years of schooling and 68.0% did not plan the pregnancy of the index child. During pregnancy, most women felt supported by the family, whereas 13.7% did not feel supported by the child’s father. Almost two thirds of the women lived with a husband or partner, and a minority (9.2%) worked outside household. In half of the sample the index child was the only child younger than 7 years living in the household. Attendance to antenatal care was almost universal (>99%), with most of the women attending ≥6 consultations and initiating antenatal care in the first trimester of pregnancy.

**Table 1:** Comparison between women with complete (analytical sample) and women with missing data (non-analytical sample).

	Non-analytical sample (*N* = 1144)	Analytical sample (*N* = 2098)	*p*^a^
	*N* (%)	*N* (%)	
Maternal age (years)			<0.001
<15	8 (0.7)	5 (0.2)	
15–19	175 (15.9)	276 (13.2)	
20–24	361 (32.8)	540 (25.7)	
25–29	242 (22.0)	506 (24.1)	
30–34	194 (17.6)	441 (21.0)	
35–39	96 (8.7)	244 (11.6)	
≥40	26 (2.4)	85 (4.1)	
Maternal skin color			<0.001
White	158 (14.4)	305 (14.6)	
Black	131 (12.0)	190 (9.1)	
Brown	756 (69.0)	1551 (74.2)	
Other	51 (4.7)	44 (2.1)	
Maternal schooling (years)			0,068
0–4	92 (8.7)	186 (9.5)	
5–8	352 (33.4)	572 (29.3)	
≥9	610 (57.9)	1192 (61.2)	
Mother planned to be pregnant (yes)	288 (26.2)	671 (32.0)	<0.001
Support from the child’s father during pregnancy (yes)	929 (84.7)	1804 (86.3)	0.223
Support from the family during pregnancy (yes)	1002 (91.4)	1948 (93.4)	0.038
Mother lives with a partner (yes)	692 (60.5)	1351 (64.4)	0.027
Mother works outside the household (yes)	117 (10.6)	192 (9.2)	0.181
Number of children <7 years of age living in the household			0.143
1	538 (47.7)	1046 (50.4)	
≥2	591 (52.3)	1031 (49.6)	
Number of antenatal care consultations			0.002
≥6	847 (78.9)	1738 (83.4)	
0–5	226 (21.1)	346 (16.6)	
First antenatal care consultation in			0.002
1st trimester	792 (73.8)	1647 (79.0)	
2nd trimester	209 (19.5)	339 (16.3)	
3rd trimester	72 (6.7)	98 (4.7)	
EPDS ≥ 10 (at T0)	309 (28.7)	532 (25.4)	0.042
EPDS ≥ 13 (at T0)	183 (17.0)	279 (13.3)	0.005

^a^Chi-square test.

In comparison to women with missing information, there was a smaller proportion of women younger than 20 years and of Black skin color in the analytical sample, and a greater proportion of women who planned the pregnancy of the index child, felt supported by the family during pregnancy, lived with a partner, attended ≥6 antenatal care consultations, and started antenatal care in the first trimester of pregnancy. Prevalence of EPDS ≥ 10 and EPDS ≥ 13 at T0 was lower in the analytical sample than among women with missing information (25.4% versus 28.7%, and 13.3% versus 17.0%, respectively).

As for children characteristics (Table 2), in the analytical sample preterm births comprised 8.4% of the sample, 6.7% were SGA, and most infants were 7–11 months of age at study entrance (mean age 7.2 months; SD = 2.9). Mean child age at T1 and T3 were, respectively, 18.7 (3.4) and 42.7 (3.5) months. Prevalence of preterm births and SGA among children from the missing sample was 8.4% and 8.7%, respectively, and most infants were 7–11 months of age at study entrance (mean age 7.5 months; SD = 2.7). Mean child age at T1 and T3 at the missing sample were, respectively, 18.6 (3.5) and 43.2 (3.3) months.

**Table 2:** Comparison between children with complete (analytical sample) and children with missing data (non-analytical sample).

	Non-analytical sample (*N* = 1144)	Analytical sample (*N* = 2098)	*p*^a^
Sex			0.257
Male	586 (51.2)	1067 (50.9)	
Female	558 (48.8)	1031 (49.1)	
Intrauterine growth			0.283
Term, AGA	817 (83.4)	1616 (85.4)	
Preterm, AGA	77 (7.9)	149 (7.9)	
Term, SGA	80 (8.2)	118 (6.2)	
Preterm, SGA	5 (0.5)	9 (0.5)	
Age at baseline (months)			0.015
0–3	132 (11.5)	319 (15.2)	
4–6	337 (29.5)	587 (28.0)	
7–11	675 (59.0)	1192 (56.8)	

^a^Chi-square test.

### Maternal depression

Mean EPDS score at T0, T1 and T3 were 6.6 (6.4–6.9), 6.4 (6.2–6.6) and 8.3 (8.2–8.5), respectively. Paired *t*-tests showed statistically significant differences between means in T0 and T1 (*p* = 0.017), T0 and T3 (*p* < 0.001), and T1 and T3 (*p* < 0.001). Supplementary Fig. 1 shows the prevalence of EPDS ≥ 10 and EPDS ≥ 13 in each follow-up in the analytical and in the multiple imputation sample.

**Fig. 2:**
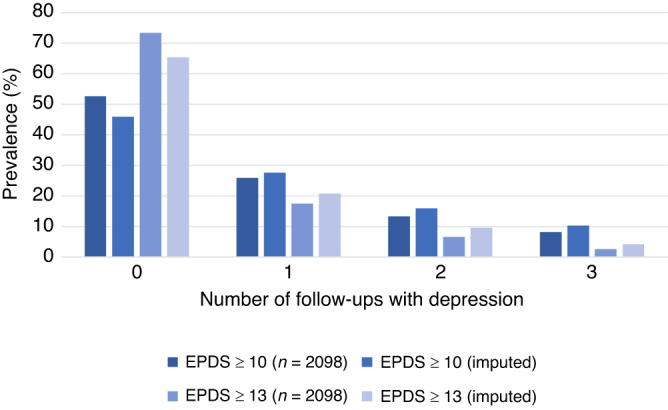
Number of follow-ups with maternal EPDS score ≥10 and ≥13 in the analytical sample (*N* = 2098) and after multiple imputation (imputed). EPDS Edinburgh Postnatal Depression Scale.

Figure 2 shows that 25.9% of the women had EPDS ≥ 10 in only one follow-up, whereas 13.4% had EPDS ≥ 10 in two follow-ups and 8.2% had persistent depressive symptoms. Multiple data imputation produced imputed prevalence of 27.6%, 16.0% and 10.4%, respectively, for women with EPDS ≥ 10 in only one, two and three follow-ups. Prevalence of EPDS ≥ 13 in only one, two and three follow-ups were, respectively, 17.5%, 6.6% and 2.6%, and after imputation the prevalence changed to 20.8%, 9.7% and 4.2%, respectively.

Supplementary Table 1 presents the number of follow-ups with EPDS ≥ 10, according, respectively, to maternal and child characteristics at T0. The prevalence of persistent EPDS ≥ 10 was more than three times higher in women with 0–4 years of schooling (18.8%; 13.8–25.1%) than in those with 9 years or more (5.8%; 4.6–7.3%). Among the women who did not feel supported by the child’s father (15.7%; 11.9–20.4%) or by the family during pregnancy (19.7%; 13.9–27.2%), the prevalence of persistent depression was twice as high than among those who felt supported the by child’s father (6.9%; 5.8–8.2%) and the family (7.3%; 6.2–8.5%). The prevalence of persistent depression was also higher among women who lived with two or more children under 7 years in the household as compared to those who lived with only the index child (9.9%; 8.8–11.9% versus 6.2%; 4.9–7.8%). After multiple imputation (Supplementary Table 2), the prevalence of EPDS ≥ 10 in three follow-ups was similar to that in the analytical sample, in addition to be higher among those living without a partner, attending less than six antenatal consultations, and initiating antenatal care later than in the first trimester of pregnancy. There was no association between number of follow-ups with EPDS ≥ 10 and the children characteristics in both the analytical (Supplementary Table 3) and the multiple imputation sample (Supplementary Table 4).

The number of follow-ups with EPDS ≥ 13 in the analytical sample, according to maternal characteristics at T0 is presented in Supplementary Table 5. The prevalence of EPDS ≥ 13 in three follow-ups was higher in older women, with lower schooling, who did not feel supported by the child’s father and the family during pregnancy, lived without a partner, and had more children under 7 years of age at the household. After multiple imputation (Supplementary Table 6), higher prevalence of EPDS ≥ 13 at three follow-ups was associated with the same variables as in the analytical sample, except maternal age (some imputations generated missing values for maternal age categories), in addition of having reported Black and other skin color, not having planned the pregnancy, attending less than six antenatal care consultations, and initiating antenatal care later than in the first trimester of pregnancy. There was no association between number of follow-ups with EPDS ≥ 13 and the children characteristics in both the analytical and the multiple imputation sample (Supplementary Tables 7 and 8).

### Maternal depression and child development

Mean ASQ3 score in the analytical sample was 220.2 (95% CI 218.0; 222.3). Supplementary Table 9 presents mean ASQ3 according to the number of follow-ups with EPDS ≥ 10 and EPDS ≥ 13 in the analytical and in the imputation sample. The ASQ3 means were very similar between the samples, regardless of the number of follow-ups with EPDS ≥ 10 and EPDS ≥ 13. The largest difference between the samples was observed in the groups with EPDS ≥ 13 in all three follow-ups, where the ASQ3 means in the analytic and imputed samples were 216.0 and 218.1, respectively—however with no statistically significant difference based on the overlap of the 95% CI.

Table 3 shows the beta coefficients for ASQ3 according to the number of follow-ups with EPDS ≥ 10. A dose-response gradient was observed in crude analysis, with a decrease of about 4, 10 and 14 points in ASQ3 score as the number of follow-ups with EPDS ≥ 10 increased from none to one, two and three, respectively (*p* for trend <0.001). After adjustment for confounders, the direction of the association persisted but lost statistical significance (*p* = 0.079), with the 95% CI of beta coefficients of all categories of the independent variable overlapping and including zero. Multiple data imputation produced an imputed estimate that was different from the available data (Table 4). In adjusted analysis, children from mothers with EPDS ≥ 10 in three follow-ups presented a decrease of about 14 points in ASQ3 (adjusted beta coefficient = −13.79; 95% CI: −22.59 to −5.00) (*p* for linear trend = 0.001).

**Table 3:** Crude and adjusted beta coefficients for ASQ3 score at T3, according to number of follow-ups with maternal EPDS ≥ 10 and EPDS ≥ 13 (*N* = 2098).

	Crude			Adjusted		
	*β*	95% CI	*p*	*β*	95% CI	*p*
Follow-ups with EPDS ≥ 10			<0.001^a^			0.079^b^
None	Ref.			Ref.		
One	−3.66	−8.80; 1.48		−5.27	−11.00; 0.45	
Two	−9.79	−16.35; −3.23		−6.48	−13.96; 1.01	
Three	−13.62	−21.69; −5.53		−9.17	−18.71; 0.38	
Follow-ups with EPDS ≥ 13			0.051^b^			0.529^b^
None	Ref.			Ref.		
One	−3.67	−9.37; 2.04		−4.43	−10.82; 1.96	
Two	−11.45	−20.19; −2.70		−3.96	−14.36; 6.44	
Three	−5.23	−18.74; 8.28		−2.69	−18.83; 13.46	

Adjusted by PCF allocation, paternal and maternal schooling, support from family during pregnancy, number of ANC visits, month in which ANC visits started, and mother working outside the household.

^a^*p* value for trend.

^b^*p* value for heterogeneity.

**Table 4:** Crude and adjusted beta coefficients for ASQ3 score at T3, according to number of follow-ups with maternal EPDS ≥ 10 and EPDS ≥ 13 (multiple imputation).

	Crude			Adjusted		
	*β*	95% CI	*p* value	*β*	95% CI	*p*
Follow-ups with EPDS ≥ 10			<0.001^a^			0.001^a^
None	Ref.			Ref.		
One	−3.39	−8.26; 1.49		−5.06	−10.56; 0.43	
Two	−9.13	−14.86; −3.39		−6.36	−13.01; 0.30	
Three	−13.97	−21.11; −6.82		−13.79	−22.59; −5.00	
Follow-ups with EPDS ≥ 13			0.011^b^			0.117^b^
None	Ref.			Ref.		
One	−4.04	−9.15; 1.06		−4.99	−10.92; 0.93	
Two	−11.79	−19.22; −4.38		−7.62	−17.00; 1.76	
Three	−3.81	−14.84; 7.21		−10.2	−24.91; 4.51	

Adjusted by PCF allocation, paternal and maternal schooling, support from family during pregnancy, number of ANC visits, month in which ANC visits started, and mother working outside the household.

^a^*p* value for trend.

^b^*p* value for heterogeneity.

In the analytical sample, there was no association between number of follow-ups with EPDS ≥ 13 and ASQ3 in crude or adjusted analyses (Table 3). In the multiple imputation data (Table 4) the number of follow-ups with EPDS ≥ 13 was associated with lower ASQ3 score only in crude analysis (*p* = 0.011) and with no evidence of trend.

## Discussion

In our study, in a 3-year postpartum period 8.2% of the women presented persistent EPDS ≥ 10. In crude analyses of the analytical sample, children from women with persistent EPDS ≥ 10 had almost 14 points less in the ASQ3 mean score than those whose mothers had no postpartum depression. This association however turned non-significant after adjustment for confounders. After multiple imputation, the crude and adjusted coefficients were statistically significant, with a dose-response gradient leading to a 14-point decrease in ASQ3 score among children from women with persistent EPDS ≥ 10, with little attenuation after adjustment for confounders. As the most disadvantaged participants and the participants more likely to experience severe depression were overrepresented in the group with missing data, these findings suggest that bias resulting from missing information was affecting the power of the study. Although statistically significant, the 14 points difference between groups is of small magnitude, representing minus 0.27 of the ASQ3 standard deviation, similar to what was found in a metanalysis of studies to evaluate the effects of maternal depressive symptoms on the cognitive development of children under 7 years of age.^13^ Worth highlighting however that the ASQ3 mean (standard deviation) in our sample was 220.2 (50.6), thus below the potential 300 points achievable in the test, with children from depressed mothers being even further behind their potential for full development.

In adjusted analyses, there was no association between persistent EPDS ≥ 13 and child development in both the analytical and multiple imputation analyses, probably due to the low prevalence of women with EPDS ≥ 13 at the three occasions (*N* = 55).

In a systematic review and metanalysis of observational studies, the prevalence of postpartum depression was higher in low-income (25.8%; 17.9–33.8%) than in the middle-income countries (20.8%; 18.4–23.1%).^24^ As our sample was composed of women of low socio-economic status living in a middle-income country, our finding of one woman in every 4 presenting EPDS ≥ 10 in at least one occasion at the postpartum period is consistent with the pooled prevalence from that metanalysis. There was an increase in depressive symptomatology from T0 to T3. Nonetheless, T3 was conducted in late 2021, during the COVID-19 pandemic. Several studies reported the impact of the pandemic on the prevalence and burden of major depressive disorder globally, with women being affected more than men: an increase of 29.8% (27.3–32.5%) compared to 24.0% (21.5–26.7%) in men.^25^ Thus, the COVID-19 pandemic might have increased the prevalence of persistent depressive symptoms in our sample.

Prevalence of persistent EPDS ≥ 10 and EPDS ≥ 13 was higher in women with lower level of education, among those who lack support from the child’s father and from the family during pregnancy, and who lived with two or more children under 7 years in the household. Prevalence of persistent EPDS ≥ 13 was also higher among older women. Other studies also have identified these as risk factors for depression.^26^

Corroborating with our current findings, in previous analysis with data from T0, children of mothers with symptoms of depression (EPDS ≥ 10) presented an adjusted reduction of ~10 points in the total ASQ-3 score (mean ASQ3 = 240.9 versus 250.7; *p* < 0.001).^27^ At the Avon Longitudinal Study of Parents and Children, EPDS was completed on 6 occasions (from 18 weeks of pregnancy up to 3 years of age), and intelligence coefficient (IQ) of the index child was measured at aged 8 years.^28^ In adjusted analyses of the imputed sample, there was no effect of prenatal or postnatal maternal depression (defined as EPDS > 12) on child cognitive development (−0.19 IQ points; −1.5; 1.1). On the other hand, the persistence of EPDS ≥ 10 at 18 and 32 weeks of gestation was associated with developmental delay at 18 months of age (adjusted OR 1.34; 1.11–1.62).^29^ Similar results were found when applying the EPDS 12/13 and 14/15 cutoffs. After further adjustment for postnatal depression, the effect sizes were slightly attenuated but remained statistically significant, thus indicating that some of the effects on child development attributed to postpartum depression are caused in part by depressive symptoms during pregnancy.^29^ Also, children of women with persistent postnatal depression of moderate (EPDS = 13–14) or marked severity (EPDS = 15–16) had about threefold increase in risk of behavioral problems at 3.5 years of age. Compared with children of women with postnatal depression that did not persist, of either moderate or severe intensity, children of women with persistent and severe (EPDS ≥ 17) depression were at an increased risk for behavioral problems by age 3.5 years as well as lower mathematics grades and depression during adolescence.^15^

Putative pathways through which maternal depression may influence children’s cognitive development include heritability of depression; innate dysfunctional neuroregulatory mechanisms; negative maternal cognitions (i.e., thoughts), behaviors, and affect; and stressful context of children’s lives.^30^ Maternal responsiveness and quality of maternal caregiving are the most important mediators through which maternal depressive symptoms are associated with children’s cognitive development in early childhood.^14^ Maternal postpartum depression is associated with a set of outcomes that are unfavorable to the proper development of the children. Depression in the general population, as well as at the postpartum period, is more frequent in families with worse socioeconomic status (mothers with less education, unemployed and with lower income) who are also exposed to poor physical health, food insecurity, domestic violence, and inadequate social support, which increase the risk for poor child neurodevelopment.^26^

### Strengths and limitations

Because our sample was composed by poor families, living in a middle-income country, and beneficiary of a cash transfer program, one limitation of this study is that the results may not apply to the general population. Also, although EPDS is among the most employed instruments to assess increased risk of depression, it is a screening tool. The instruments used for measuring depressive symptoms and child development are useful as general screeners and cannot replace a diagnostic interview to identify cases of clinical disorders. As a result, we could not verify which women may have had clinical depressive disorders. A further limitation is that we lack information on maternal depression in the antenatal period. In addition to new challenges associated with parenting, postnatal depression might be affected by continuity of earlier depression.^26^

Several strengths distinguish this study. First, we relied on well-established, validated instruments for measuring depressive symptoms and child development. Second, child development was assessed by observation of task completion by the interviewers (previous studies relied on maternal report for measures of infant outcomes and depressed mothers may be more critical or less observant of their child’s development). Third, the longitudinal design of the study allowed assessment of maternal depression in three postpartum periods before developmental outcome was assessed. This avoided assessing exposures based on recall or confusing the temporal sequence of the exposure and outcome. Fourth, the large sample size and data imputation allowed for fairly precise estimates across the exposure distribution. And finally, the study corroborates the limited existing evidence on the impact of persistent postpartum depression on child development and sheds light on this association in a poor population in a middle-income country, as opposed to existing research conducted in high-income countries.

### Conclusions and recommendations

In this longitudinal study of poor families, beneficiaries of a cash transfer program in Brazil, we found that almost one in every ten women presented persistent depression across the first 3 years postpartum. A detrimental impact of persistent maternal depression symptoms on child development at 3 years of age was observed. As maternal depressive symptoms are often common in pregnancy and continue through to the postnatal period, it is worrying that, even attending several antenatal care consultations, and starting antenatal care early in pregnancy, a large proportion of women presented depressive symptoms in the first year that extended until the third year postpartum. Due to the high prevalence and potential for impairing child development, identification of women at increased risk of depression should be among the primary health care sector priorities in maternal and child health in Brazil.

### Supplementary Information

Checklist

Supplementary Material

## Data Availability

All study data are publicly available for use by other research teams wishing to use them for non-commercial purposes, without breaching participant confidentiality.
