# Supplementary material for: Maternal depression and child development at 3 years of age: a longitudinal study in a Brazilian child development promotion program
Source: Pediatr Res. 2023 Nov 11;95(4):1139–46. doi: 10.1038/s41390-023-02876-9 (PMC10920190; doi:10.1038/s41390-023-02876-9)
Supplement: Supplementary file 2 — Supplementary Material [file 41390_2023_2876_MOESM2_ESM.pdf]

SUPPLEMENTARY MATERIAL

Maternal depression and child development at 3 years of age: A longitudinal study

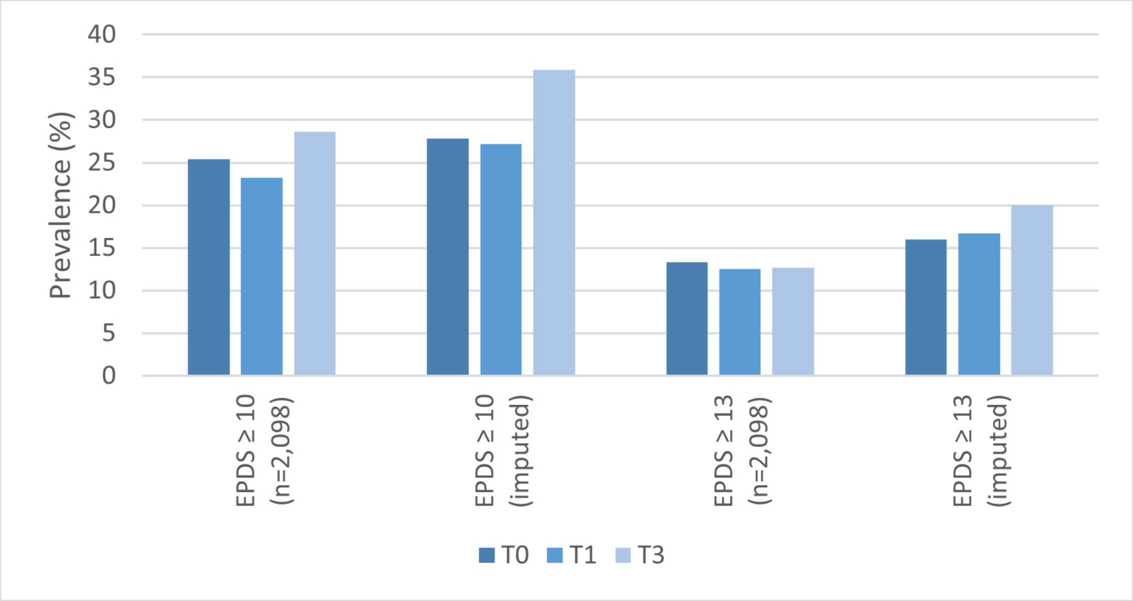

Supplementary Figure 1. Prevalence of EPDS ≥10 and EPDS ≥13 in each follow-up in the analytical sample (N=2,098) and after multiple imputation (imputed)

Supplementary Table 1. Proportion of women with none, one, two or three follow-up visits with EPDS ≥10, according to independent characteristics (N= 2,098)

Variables	N (%)	Number of follow-ups with EPDS ≥ 10 % (95%CI) 0	1	2	3	p*
		% (IC95%)	% (IC95%)	% (IC95%)	% (IC95%)	
**Maternal age (years)**						0,001
< 15	5 (0.2)	40.0 (10.0; 80.0)	20.0 (2.7; 69.1)	40.0 (10.0; 80.0)	0,0	
15-19	276 (13.2)	60.9 (55.0; 66.5)	25.0 (20.2; 30.4)	10.9 (7.7; 15.1)	3.3 (1.7; 6.1)	
20-24	540 (25.7)	56.3 (52.1; 60.4)	24.4 (21.0; 28.3)	12.8 (10.2; 15.9)	6.5 (4.7; 8.9)	
25-29	506 (24.1)	51.4 (47.0; 55.7)	26.3 (22.6; 30.3)	13.8 (11.1; 17.1)	8.5 (6.4; 11.3)	
30-34	441 (21.0)	50.6 (45.9; 55.2)	24.5 (20.7; 28.7)	13.8 (10.9; 17.4)	11.1 (8.5; 14.4)	
35-39	244 (11.6)	46.7 (40.5; 53.0)	27.5 (22.2; 33.4)	16.0 (11.9; 21.1)	9.8 (6.7; 14.3)	
≥ 40	85 (4.1)	36.5 (27.0; 47.2)	38.8 (29.1; 49.5)	11.8 (6.4; 20.5)	12.9 (7.3; 21.9)	
**Maternal skin color**						0,165
White	305 (14.6)	53.4 (47.8; 59.0)	28.5 (23.7; 33.9)	12.1 (8.9; 16.3)	5.9 (3.7; 9.2)	
Black	190 (9.1)	50.5 (43.5; 57.6)	22.6 (17.2; 29.1)	16.3 (11.7; 22.3)	10.5 (6.9; 15.8)	
Brown	1,551 (74.2)	52.3 (49.8; 54.8)	25.8 (23.7; 28.0)	13.5 (11.9; 15.3)	8.4 (7.1; 9.9)	
Other	44 (2.1)	70.5 (55.5; 82.0)	18.2 (9.4; 32.4)	6.8 (2.2; 19.1)	4.5 (1.1; 16.4)	
**Maternal schooling (years)**						< 0.001
0-4	186 (9.5)	41.4 (34.5; 48.6)	22.6 (17.1; 29.1)	17.2 (12.4; 23.3)	18.8 (13.8; 25.1)	
5-8	572 (29.3)	47.0 (43.0; 51.1)	28.1 (24.6; 32.0)	15.2 (12.5; 18.4)	9.6 (7.5; 12.3)	
≥ 9	1,192 (61.2)	57.7 (54.9; 60.5)	24.8 (22.5; 27.4)	11.7 (10.0; 13.6)	5.8 (4.6; 7.3)	
**Mother planned to be pregnant?**						0,013
No	1,424 (68.0)	50.3 (47.7; 52.9)	26.5 (24.2; 28.8)	14.6 (12.9; 16.5)	8.6 (7.3; 10.2)	
Yes	671 (32.0)	57.4 (53.6; 61.1)	24.6 (21.5; 28.0)	10.9 (8.7; 13.5)	7.2 (5.4; 9.4)	
**Support from the father during pregnancy?**						< 0.001

No	287 (13.7)	42.5 (36.9; 48.3)	26.8 (22.0; 32.3)	15.0 (11.3; 19.6)	15.7 (11.9; 20.4)	
Yes	1,804 (86.3)	54.2 (51.9; 56.5)	25.7 (23.8; 27.8)	13.1 (11.7; 14.8)	6.9 (5.8; 8.2)	
**Support from the family during pregnancy?**						< 0.001
No	137 (6.6)	32.8 (25.5; 41.1)	25.5 (18.9; 33.5)	21.9 (15.8; 29.6)	19.7 (13.9; 27.2)	
Yes	1,948 (93.4)	54.2 (51.9; 56.4)	25.9 (24.0; 27.9)	12.7 (11.3; 14.2)	7.3 (6.2; 8.5)	
**Lives with partner**						0,117
No	746 (35.6)	49.7 (46.1; 53.3)	26.3 (23.2; 29.6)	14.3 (12.0; 17.0)	9.7 (7.7; 12.0)	
Yes	1,351 (64.4)	54.1 (51.4; 56.8)	25.7 (23.4; 28.1)	12.9 (11.2; 14.8)	7.3 (6.1; 8.8)	
**Works outside the household**						0,759
No	1,905 (90.8)	52.8 (50.6; 55.0)	25.7 (23.8; 27.7)	13.5 (12.0; 15.1)	8.0 (6.8; 9.3)	
Yes	192 (9.2)	50.5 (43.5; 57.5)	27.1 (21.3; 33.8)	12.5 (8.5; 18.0)	9.9 (6.4; 15.0)	
**Number of children < 7 years of age living in the household**						< 0.001
1	1,046 (50.4)	56.7 (53.7; 59.7)	26.3 (23.7; 29.0)	10.8 (9.1; 12.8)	6.2 (4.9; 7.8)	
≥ 2	1,031 (49.6)	48.9 (45.8; 51.9)	25.2 (22.7; 28.0)	16.0 (13.9; 18.4)	9.9 (8.2; 11.9)	
**Number of antenatal care consultations**						0,099
≥ 6	1,738 (83.4)	53.7 (51.4; 56.1)	25.4 (23.4; 27.5)	12.9 (11.4; 14.6)	7.9 (6.8; 9.3)	
0-5	346 (16.6)	46.5 (41.3; 51.8)	28.3 (23.8; 33.3)	15.9 (12.4; 20.1)	9.2 (6.6; 12.8)	
**First antenatal care visit in**						0,303
1st trimester	1,647 (79.0)	53.8 (51.4; 56.2)	25.5 (23.5; 27.7)	12.6 (11.1; 14.3)	8.1 (6.9; 9.6)	
2nd trimester	339 (16.3)	48.7 (43.4; 54.0)	26.8 (22.4; 31.8)	16.5 (12.9; 20.9)	8.0 (5.5; 11.4)	
3rd trimester	98 (4.7)	44.9 (35.4; 54.8)	28.6 (20.5; 38.3)	17.3 (11.1; 26.2)	9.2 (4.8; 16.7)	

*p value of heterogeneity

Supplementary Table 2. Proportion of women with none, one, two or three follow-up visits with EPDS ≥10, according to independent characteristics (multiple imputation)

	Number of follow-ups with EPDS ≥ 10 % (95%CI)				
	**0**	**1**	**2**	**3**	**p***
**Maternal age (years)****					-
< 15					
15-19					
20-24					
25-29					
30-34					
35-39					
≥ 40					
**Maternal skin color**					0,065
White	48.9 (44.1; 53.8)	29.6 (25.1; 34.1)	13.6 (10.2; 16.9)	7.9 (5.4; 10.4)	
Black	42.5 (36.8; 48.3)	25.9 (20.6; 31.1)	18.2 (13.6; 22.7)	13.4 (9.4; 17.5)	
Brown	46.7 (44.5; 48.8)	27.5 (25.5; 29.4)	16.3 (14.7; 17.9)	9.6 (8.3; 10.9)	
Other	49.5 (38.8; 60.2)	27.9 (17.8; 38.0)	12.4 (4.9; 20.0)	10.2 (3.9; 16.5)	
**Maternal schooling (years)**					< 0.001
0-4	36.9 (31.0; 42.8)	23.4 (18.1; 28.8)	19.7 (14.8; 24.7)	19.9 (15.1; 24.8)	
5-8	41.7 (38.3; 45.1)	28.5 (25.3; 31.8)	17.7 (15.0; 20.4)	12.1 (9.8; 14.3)	
≥ 9	51.5 (49.1; 53.9)	27.6 (25.3; 29.8)	13.8 (12.1; 15.6)	7.1 (5.8; 8.3)	
**Mother planned to be pregnant?**					< 0.001
No	44.7 (42.5; 46.8)	28.1 (26.1; 30.2)	16.7 (15.1; 18.4)	10.5 (9.2; 11.9)	
Yes	51.0 (47.7; 54.3)	26.9 (23.9; 30.0)	14.0 (11.5; 16.4)	8.1 (6.3; 9.9)	
**Support from the father during pregnancy?**					< 0.001
No	34.9 (30.3; 39.4)	28.2 (23.8; 32.5)	17.5 (13.8; 21.2)	19.5 (15.7; 23.2)	

Yes	48.6 (46.6; 50.6)	27.7 (25.8; 29.5)	15.6 (14.1; 17.1)	8.2 (7.1; 9.3)	
**Support from the family during pregnancy?**					< 0.001
No	28.6 (22.5; 34.8)	25.4 (19.3; 31.5)	23.1 (17.3; 29.0)	22.9 (17.1; 28.6)	
Yes	48.1 (46.2; 50.1)	27.9 (26.2; 29.7)	15.2 (13.8; 16.6)	8.7 (7.6; 9.8)	
**Lives with partner**					< 0.001
No	41.1 (38.1; 44.0)	28.8 (26.0; 31.5)	17.9 (15.6; 20.2)	12.3 (10.4; 14.3)	
Yes	48.8 (46.5; 51.1)	27.0 (24.9; 29.1)	15.0 (13.3; 16.6)	9.2 (7.9; 10.6)	
**Works outside the household**					0,48
No	46.9 (44.9; 48.8)	27.5 (25.8; 29.3)	15.8 (14.4; 17.3)	9.8 (8.6; 10.9)	
Yes	43.9 (38.1; 49.8)	30.0 (24.5; 35.5)	16.3 (11.8; 20.8)	9.8 (6.3; 13.3)	
**Number of children < 7 years of age living in the household**					< 0.001
1	49.8 (47.2; 52.4)	27.9 (25.5; 30.3)	14.0 (12.2; 15.9)	8.2 (6.8; 9.7)	
≥ 2	42.8 (40.2; 45.3)	27.3 (24.9; 29.6)	17.7 (15.7; 19.7)	12.3 (10.6; 14.0)	
**Number of antenatal care consultations**					0,001
≥ 6	48.3 (46.3; 50.3)	27.3 (25.5; 29.2)	15.3 (13.8; 16.8)	9.1 (7.9; 10.3)	
0-5	40.5 (36.2; 44.8)	29.7 (25.5; 33.9)	18.0 (14.6; 21.4)	11.8 (9.0; 14.6)	
**First antenatal care visit in**					0,001
1st trimester	48.5 (46.4; 50.6)	27.3 (25.4; 29.2)	15.0 (13.4; 16.5)	9.2 (8.0; 10.4)	
2nd trimester	43.4 (38.9; 47.9)	28.3 (24.1; 32.5)	17.6 (14.2; 21.0)	10.7 (7.9; 13.4)	
3rd trimester	34.6 (27.1; 42.1)	32.8 (25.1; 40.4)	21.3 (14.5; 28.1)	11.4 (6.3; 16.4)	

*Chi-square test; **Some imputations generated missing values for maternal age categories

Supplementary Table 3. Proportion of women with none, one, two or three follow-up visits with EPDS ≥10, according to children characteristics. (N= 2,098)

	N (%)	Number of follow-ups with EPDS ≥ 10 % (95%CI)				
		**0**	**1**	**2**	**3**	**p***
**Sex**						0,489
Female	1,067 (50.9)	51.5 (48.5; 54.5)	27.3 (24.7; 30.0)	12.9 (11.0; 15.1)	8.2 (6.7; 10.1)	
Male	1,031 (49.1)	53.6 (50.6; 56.7)	24.4 (21.9; 27.2)	13.9 (11.9; 16.1)	8.1 (6.5; 9.9)	
**Age at first follow-up (months)**						0,079
0-3	319 (15.2)	58.9 (53.4; 64.2)	21.9 (17.7; 26.8)	13.2 (9.9; 17.3)	6.0 (3.8; 9.2)	
4-6	587 (28.0)	51.6 (47.6; 55.6)	28.6 (25.1; 32.4)	12.6 (10.2; 15.5)	7.2 (5.3; 9.5)	
7-12	1,192 (56.8)	51.3 (48.5; 54.2)	25.6 (23.2; 28.1)	13.8 (12.0; 15.9)	9.2 (7.7; 11.0)	
**Intrauterine growth**						0,785
Term, AGA	1,616 (85.4)	52.8 (50.3; 55.2)	26.1 (24.0; 28.3)	12.9 (11.4; 14.7)	8.2 (6.9; 9.6)	
Preterm, AGA	149 (7.9)	50.3 (42.4; 58.3)	27.5 (20.9; 35.2)	16.8 (11.6; 23.7)	5.4 (2.7; 10.4)	
Term, SGA	118 (6.2)	50.8 (41.9; 59.7)	26.3 (19.1; 34.9)	16.1 (10.5; 23.9)	6.8 (3.4; 13.0)	
Preterm, SGA	9 (0.5)	44.4 (17.7; 74.9)	33.3 (11.1; 66.7)	22.2 (5.6; 57.9)	0.0	

*Chi-square test

Supplementary Table 4. Proportion of women with none, one, two or three follow-up visits with EPDS ≥10, according to children characteristics (multiple imputation)

	Number of follow-ups with EPDS ≥ 10<br>% (95%CI)				
	**0**	**1**	**2**	**3**	**p***
**Sex**					0,459
Female	46.2 (43.6; 48.8)	28.4 (26.1; 30.8)	15.3 (13.4; 17.1)	10.1 (8.6; 11.6)	
Male	45.7 (43.1; 48.2)	26.9 (24.5; 29.2)	16.8 (14.8; 18.8)	10.7 (9.1; 12.3)	
**Age at first follow-up (months)**					0,244
0-3	49.2 (44.5; 54.0)	25.0 (20.8; 29.2)	16.9 (13.3; 20.6)	8.8 (6.1; 11.6)	
4-6	45.9 (42.5; 49.4)	29.7 (26.5; 32.9)	14.7 (12.3; 17.1)	9.6 (7.7; 11.6)	
7-12	45.1 (42.8; 47.5)	27.3 (25.1; 29.5)	16.5 (14.6; 18.3)	11.1 (9.6; 12.6)	
**Intrauterine growth**					0,944
Term, AGA	47.0 (44.9; 49.1)	27.6 (25.7; 29.6)	15.5 (13.9; 17.0)	10.0 (8.7; 11.2)	
Preterm, AGA	45.5 (38.7; 52.3)	28.3 (22.0; 34.6)	17.8 (12.5; 23.1)	8.5 (4.7; 12.2)	
Term, SGA	44.5 (37.1; 51.8)	28.9 (22.1; 35.8)	16.7 (11.2; 22.1)	9.9 (5.6; 14.2)	
Preterm, SGA	45.2 (17.3; 73.0)	26.0 (1.0; 51.1)	28.8 (4.9; 52.6)	0.0	

*Chi-square test

Supplementary Table 5. Proportion of women with none, one, two or three follow-up visits with EPDS ≥13, according to independent characteristics (N= 2,098)

	N (%)	Number of follow-ups with EPDS ≥13 % (95%CI)				
		**0**	**1**	**2**	**3**	**p***
**Maternal age (years)**						0,026
< 15	5 (0.2)	60.0 (20.0; 90.0)	20.0 (2.7; 69.1)	20.0 (2.7; 69.1)	0.0	
15-19	276 (13.2)	80.1 (74.9; 84.4)	14.5 (10.8; 19.2)	4.3 (2.5; 7.5)	1.1 (0.4; 3.3)	
20-24	540 (25.7)	76.3 (72.5; 79.7)	16.3 (13.4; 19.7)	5.6 (3.9; 7.8)	1.9 (1.0; 3.4)	
25-29	506 (24.1)	72.3 (68.3; 76.1)	18.0 (14.9; 21.6)	6.9 (5.0; 9.5)	2.8 (1.6; 4.6)	
30-34	441 (21.0)	70.3 (65.9; 74.4)	18.8 (15.4; 22.7)	8.2 (5.9; 11.1)	2.7 (1.6; 4.7)	
35-39	244 (11.6)	70.9 (64.9; 76.3)	19.3 (14.8; 24.7)	5.3 (3.1; 9.0)	4.5 (2.5; 8.0)	
≥ 40	85 (4.1)	60.0 (49.3; 69.8)	21.2 (13.8; 31.1)	12.9 (7.3; 21.9)	5.9 (2.5; 13.4)	
**Maternal skin color**						0,107
White	305 (14.6)	75.1 (69.9; 79.6)	17.7 (13.8; 22.4)	5.2 (3.2; 8.4)	2.0 (0.9; 4.3)	
Black	190 (9.1)	65.3 (58.2; 71.7)	20.5 (15.4; 26.9)	10.0 (6.5; 15.1)	4.2 (2.1; 8.2)	
Brown	1,551 (74.2)	73.6 (71.3; 75.7)	17.3 (15.5; 19.3)	6.4 (5.3; 7.8)	2.6 (2.0; 3.6)	
Other	44 (2.1)	86.4 (72.8; 93.7)	6.8 (2.2; 19.1)	6.8 (2.2; 19.1)	0.0	
**Maternal schooling (years)**						< 0.001
0-4	186 (9.5)	60.8 (53.6; 67.5)	20.4 (15.2; 26.8)	15.1 (10.6; 20.9)	3.8 (1.8; 7.7)	
5-8	572 (29.3)	68.4 (64.4; 72.0)	21.3 (18.2; 24.9)	5.9 (4.3; 8.2)	4.4 (3.0; 6.4)	
≥ 9	1,192 (61.2)	77.6 (75.1; 79.9)	15.7 (13.7; 17.9)	5.1 (4.0; 6.5)	1.6 (1.0; 2.5)	
**Mother planned to be pregnant?**						0,092
No	1,424 (68.0)	71.6 (69.2; 73.8)	18.6 (16.7; 20.7)	7.0 (5.7; 8.4)	2.9 (2.1; 3.9)	
Yes	671 (32.0)	76.8 (73.4; 79.8)	15.4 (12.8; 18.3)	5.8 (4.3; 7.9)	2.1 (1.2; 3.5)	
**Support from the father during pregnancy?**						< 0.001
No	287 (13.7)	60.3 (54.5; 65.8)	22.0 (17.5; 27.1)	11.5 (8.3; 15.7)	6.3 (4.0; 9.7)	

Yes	1,804 (86.3)	75.3 (73.3; 77.3)	16.9 (15.2; 18.7)	5.8 (4.8; 7.0)	2.0 (1.4; 2.8)	
**Support from the family during pregnancy?**						< 0.001
No	137 (6.6)	59.1 (50.7; 67.0)	18.2 (12.6; 25.6)	13.1 (8.4; 19.9)	9.5 (5.6; 15.7)	
Yes	1,948 (93.4)	74.3 (72.3; 76.2)	17.5 (15.8; 19.2)	6.1 (5.1; 7.3)	2.1 (1.6; 2.8)	
**Lives with partner**						< 0.001
No	746 (35.6)	70.1 (66.7; 73.3)	17.8 (15.2; 20.7)	7.5 (5.8; 9.6)	4.6 (3.3; 6.3)	
Yes	1,351 (64.4)	75.0 (72.6; 77.2)	17.4 (15.5; 19.5)	6.1 (4.9; 7.5)	1.6 (1.0; 2.4)	
**Works outside the household**						0,386
No	1,905 (90.8)	73.6 (71.6; 75.6)	17.4 (15.8; 19.2)	6.5 (5.4; 7.7)	2.5 (1.9; 3.3)	
Yes	192 (9.2)	69.3 (62.4; 75.4)	18.8 (13.8; 24.9)	7.8 (4.8; 12.6)	4.2 (2.1; 8.1)	
**Number of children < 7 years of age living in the household**						0,001
1	1,046 (50.4)	77.2 (74.5; 79.6)	16.0 (13.9; 18.3)	5.0 (3.8; 6.5)	1.9 (1.2; 2.9)	
≥ 2	1,031 (49.6)	70.0 (67.2; 72.8)	18.4 (16.2; 20.9)	8.2 (6.7; 10.1)	3.3 (2.4; 4.6)	
**Number of antenatal care consultations**						0,419
≥ 6	1,738 (83.4)	73.7 (71.6; 75.7)	17.3 (15.6; 19.2)	6.6 (5.5; 7.9)	2.4 (1.7; 3.2)	
0-5	346 (16.6)	71.1 (66.1; 75.6)	18.8 (15.0; 23.3)	6.4 (4.2; 9.5)	3.8 (2.2; 6.4)	
**First antenatal care visit in**						0,209
1st trimester	1,647 (79.0)	73.7 (71.5; 75.8)	17.7 (16.0; 19.7)	6.0 (4.9; 7.2)	2.6 (1.9; 3.5)	
2nd trimester	339 (16.3)	71.4 (66.3; 75.9)	16.8 (13.2; 21.2)	8.6 (6.0; 12.0)	3.2 (1.8; 5.8)	
3rd trimester	98 (4.7)	72.4 (62.8; 80.4)	17.3 (11.1; 26.2)	10.2 (5.6; 17.9)	0.0	

*Chi-squre test

Supplementary Table 6. Proportion of women with none, one, two or three follow-up visits with EPDS ≥13, according to independent characteristics (multiple imputation)

	Number of follow-ups with EPDS ≥ 13 % (95%CI)				
	**0**	**1**	**2**	**3**	**p***
**Maternal age (years)****					-
< 15					
15-19					
20-24					
25-29					
30-34					
35-39					
≥ 40					
**Maternal skin color**					0,003
White	69.7 (65.4; 74.0)	20.2 (16.3; 24.0)	7.3 (4.8; 9.8)	2.8 (1.2; 4.3)	
Black	57.9 (52.3; 63.4)	23.8 (18.9; 28.7)	13.0 (9.1; 16.9)	5.3 (2.7; 7.9)	
Brown	66.6 (64.6; 68.6)	20.6 (18.9; 22.4)	9.3 (8.1; 10.6)	3.5 (2.7; 4.2)	
Other	69.6 (60.0; 79.3)	16.1 (8.0; 24.1)	8.2 (2.1; 14.3)	6.0 (0.7; 11.3)	
**Maternal schooling (years)**					< 0.001
0-4	54.3 (48.4; 60.3)	21.8 (16.8; 26.8)	17.7 (13.0; 22.3)	6.2 (3.2; 9.1)	
5-8	62.6 (59.4; 65.8)	22.5 (19.6; 25.3)	9.5 (7.4; 11.5)	5.5 (3.9; 7.0)	
≥ 9	70.8 (68.7; 73.0)	19.7 (17.8; 21.7)	7.2 (6.0; 8.5)	2.2 (1.5; 3.0)	
**Mother planned to be pregnant?**					0,001
No	64.6 (62.5; 66.6)	21.5 (19.7; 23.3)	9.8 (8.5; 11.1)	4.2 (3.3; 5.1)	
Yes	70.1 (67.1; 73.1)	19.3 (16.7; 22.0)	8.2 (6.4; 10.1)	2.3 (1.3; 3.3)	
**Support from the father during pregnancy?**					< 0.001

No	49.3 (44.7; 54.0)	25.6 (21.4; 29.7)	15.4 (11.9; 18.8)	9.7 (6.9; 12.5)	
Yes	69.1 (67.3; 70.9)	20.0 (18.4; 21.6)	8.3 (7.2; 9.4)	2.6 (1.9; 3.2)	
**Support from the family during pregnancy?**					< 0.001
No	52.4 (45.7; 59.1)	20.0 (14.4; 25.7)	17.0 (11.7; 22.2)	10.7 (6.4; 14.9)	
Yes	67.4 (65.7; 69.2)	20.9 (19.3; 22.4)	8.7 (7.6; 9.7)	3.1 (2.4; 3.7)	
**Lives with partner**					< 0.001
No	58.9 (56.1; 61.8)	23.0 (20.5; 25.5)	12.0 (10.1; 13.9)	6.1 (4.7; 7.4)	
Yes	69.1 (67.0; 71.1)	19.5 (17.7; 21.3)	8.3 (7.0; 9.6)	3.1 (2.3; 3.9)	
**Works outside the household**					0,135
No	66.6 (64.9; 68.4)	20.5 (19.0; 22.1)	9.2 (8.1; 10.3)	3.6 (2.9; 4.3)	
Yes	62.0 (56.5; 67.6)	23.9 (18.8; 28.9)	10.4 (6.8; 14.0)	3.7 (1.5; 5.8)	
**Number of children < 7 years of age living in the household**					< 0.001
1	68.9 (66.5; 71.2)	19.8 (17.7; 21.8)	7.8 (6.4; 9.2)	3.5 (2.6; 4.5)	
≥ 2	62.7 (60.3; 65.2)	21.3 (19.2; 23.4)	11.2 (9.6; 12.8)	4.8 (3.7; 5.9)	
**Number of antenatal care consultations**					0,011
≥ 6	67.6 (65.7; 69.4)	20.3 (18.7; 22.0)	8.9 (7.7; 10.1)	3.2 (2.5; 3.9)	
0-5	62.1 (58.0; 66.2)	22.9 (19.2; 26.5)	10.5 (7.8; 13.2)	4.5 (2.7; 6.4)	
**First antenatal care visit in**					0,015
1st trimester	67.8 (65.9; 69.7)	20.4 (18.7; 22.0)	8.4 (7.3; 9.6)	3.4 (2.7; 4.2)	
2nd trimester	63.8 (59.6; 67.9)	21.5 (17.8; 25.1)	10.8 (8.1; 13.6)	3.9 (2.2; 5.7)	
3rd trimester	58.4 (50.6; 66.1)	24.8 (17.9; 31.8)	14.8 (9.2; 20.4)	2.0 (-0.3; 4.3)	

*Chi-square test; ** Some imputations generated missing values for maternal age categories

Supplementary Table 7. Proportion of women with none, one, two or three follow-up visits with EPDS ≥ 13, according to children characteristics (N= 2,098)

	N (%)	Number of follow-ups with EPDS ≥ 13 % (95%CI)				
		0	1	2	3	p*
**Sex**						0,962
Female	1,067 (50.9)	73.8 (71.0; 76.3)	17.2 (15.1; 19.6)	6.5 (5.1; 8.1)	2.5 (1.7; 3.7)	
Male	1,031 (49.1)	72.7 (69.9; 75.4)	17.8 (15.6; 20.3)	6.7 (5.3; 8.4)	2.7 (1.9; 3.9)	
**Age at first follow-up (months)**						0,371
0-3	319 (15.2)	78.1 (73.2; 82.3)	14.4 (11.0; 18.7)	4.7 (2.9; 7.7)	2.8 (1.5; 5.3)	
4-6	587 (28.0)	73.6 (69.9; 77.0)	16.9 (14.0; 20.1)	7.2 (5.3; 9.5)	2.4 (1.4; 4.0)	
7-12	1,192 (56.8)	71.8 (69.2; 74.3)	18.7 (16.6; 21.0)	6.8 (5.5; 8.4)	2.7 (1.9; 3.8)	
**Intrauterine growth**						0,475
Term, AGA	1,616 (85.4)	74.0 (71.8; 76.1)	16.7 (15.0; 18.6)	6.6 (5.5; 7.9)	2.7 (2.0; 3.6)	
Preterm, AGA	149 (7.9)	68.5 (60.6; 75.4)	22.8 (16.8; 30.2)	6.0 (3.2; 11.2)	2.7 (1.0; 6.9)	
Term, SGA	118 (6.2)	72.0 (63.3; 79.4)	20.3 (14.0; 28.6)	5.9 (2.9; 11.9)	1.7 (0.4; 6.5)	
Preterm, SGA	9 (0.5)	66.7 (33.3; 88.9)	11.1 (1.5; 50.0)	22.2 (5.6; 57.9)	0.0	

*Chi-square test

Supplementary Table 8. Proportion of women with none, one, two or three follow-up visits with EPDS ≥13, according to children characteristics (multiple imputation)

	Number of follow-ups with EPDS ≥ 13 % (95%CI)				
	**0**	**1**	**2**	**3**	**p***
**Sex**					0,124
Female	66.5 (64.2; 68.9)	20.5 (18.4; 22.6)	9.2 (7.7; 10.7)	3.8 (2.8; 4.8)	
Male	64.1 (61.7; 66.6)	21.1 (19.0; 23.2)	10.1 (8.6; 11.6)	4.6 (3.6; 5.7)	
**Age at first follow-up (months)**					0,475
0-3	66.7 (62.3; 71.1)	19.2 (15.5; 23.0)	9.8 (6.9; 12.6)	4.3 (2.3; 6.2)	
4-6	66.6 (63.5; 69.7)	20.0 (17.3; 22.8)	9.4 (7.4; 11.4)	4.0 (2.7; 5.3)	
7-12	64.4 (62.1; 66.6)	21.6 (19.6; 23.5)	9.7 (8.3; 11.2)	4.3 (3.3; 5.3)	
**Intrauterine growth**					0,807
Term, AGA	66.9 (64.9; 68.8)	20.0 (18.4; 21.7)	9.3 (8.0; 10.5)	3.8 (3.0; 4.6)	
Preterm, AGA	64.6 (58.2; 71.0)	21.5 (15.8; 27.2)	8.7 (4.8; 12.6)	5.2 (2.2; 8.2)	
Term, SGA	64.1 (57.2; 71.0)	22.8 (16.7; 29.0)	9.2 (5.0; 13.5)	3.8 (1.1; 6.6)	
Preterm, SGA	63.1 (37.2; 88.9)	15.5 (-4.1; 35.2)	21.4 (-0.1; 42.9)	0.0	

*Chi-square test

Supplementary Table 9. ASQ3 mean in analytic (not imputed) and imputed sample according to the number of follow-ups with EPDS ≥10 and EPDS ≥13

	Analytic sample (no imputation)			Imputed sample		
	**N**	**ASQ3 mean**	**95%CI**	**N**	**ASQ3 mean**	**95% CI**
**Total**	2,098	220.2	218.0; 222.3	3,032	220.0	218.0; 222.0
**Follow-ups with EPDS ≥10**						
None		223.6	220.7; 226.5		223.6	220.8; 226.4
One		220.1	215.9; 224.3		220.1	216.3; 224.0
Two		214.0	207.7; 220.3		214.4	209.2; 219.6
Three		209.5	202.0; 217.0		209.4	202.6; 216.3
**Follow-ups with EPDS ≥13**						
None		221.7	219.1; 224.2		221.8	219.5; 224.2
One		218.3	213.4; 223.3		217.9	213.4; 222.3
Two		209.8	200.6; 218.9		209.8	202.4; 217.2
Three		216.0	202.4; 229.6		218.1	206.8; 229.5
